# Depression before and after diagnostic procedures among women with abnormal finding of Papanicolaou screening test

**DOI:** 10.1002/cam4.4709

**Published:** 2022-03-24

**Authors:** Irena Ilic, Goran Babic, Aleksandra Dimitrijevic, Sandra Sipetic Grujicic, Milena D. Ilic

**Affiliations:** ^1^ Faculty of Medicine University of Belgrade Belgrade Serbia; ^2^ Department of Gynecology and Obstetrics, Faculty of Medical Sciences University of Kragujevac Kragujevac Serbia; ^3^ Institute of Epidemiology, Faculty of Medicine University of Belgrade Belgrade Serbia; ^4^ Department of Epidemiology, Faculty of Medical Sciences University of Kragujevac Kragujevac Serbia

**Keywords:** cervical cancer screening, depression, diagnostic procedures, Papanicolaou smear

## Abstract

**Background:**

Some studies did find significant differences in the level of depression of women while undergoing diagnostic evaluation of an abnormal Papanicolaou screening smear, but findings were not consistent. This study aimed to assess prevalence and correlates of depression in women with abnormal cervical screening results before and after diagnostic procedures.

**Methods:**

A cross‐sectional study was carried out during 2017 in a cohort of women with positive Papanicolaou screening test before and after diagnostic procedures (colposcopy/biopsy/endocervical curettage) at the university Clinical Centre Kragujevac, Serbia. Women completed a questionnaire about demographics, lifestyle, and other factors of interest. Also, questionnaire “Hospital Anxiety and Depression Scale” (HADS) was used immediately before and 2–4 weeks after the diagnostic procedures: a score of ≥8 on HADS‐D and HADS‐A subscales indicated depression and anxiety, respectively. Multivariate logistic regression was applied in the data analysis.

**Results:**

The study comprised 172 women, giving a response rate of 72.3%. The mean age of the participants was 47.8 ± 11.1 years (range 23–65). The frequency of depressive symptoms was significantly higher after diagnostic procedures (48.3%) than before diagnostic procedures (37.2%) (*p* = 0.038). Before diagnostic procedures, older age (OR = 1.60; 95% CI = 1.09–2.34; *p* = 0.017), and level of anxiety according to the HADS‐A subscale (OR = 1.61; 95% CI = 1.38–1.88; *p* < 0.001) were significant independent predictors of depression. After diagnostic procedures, significant independent predictors of depression were urban place of residence (OR = 0.12; 95% CI = 0.03–0.47; *p* = 0.002) and level of anxiety according to the HADS‐A subscale (OR = 1.85; 95% CI = 1.54–2.21; *p* < 0.001).

**Conclusion:**

Our study showed that older age, rural residence, and anxiety play a role in shaping the risk of depression among women undergoing additional diagnostic procedures after receiving an abnormal Papanicolaou screening result.

## Graphical abstract

Women experience significant emotional distress while undergoing further diagnostic evaluation of an abnormal Papanicolaou smear. But, knowledge about the factors that contribute to depressive symptoms before and after diagnostic procedures is insufficient. This study aimed to assess prevalence and correlates of depression in women with abnormal cervical screening results before and after diagnostic procedures. #10;Almost half of the women in our population experienced elevated depressive symptoms while undergoing the cervical cancer screening, particularly those of older age, with rural place of residence and higher level of anxiety. Findings from this study may inform health authorities about the possibilities to improve adherence to follow‐up in cervical cancer screening program.

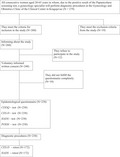

## 1 INTRODUCTION

In developed countries, a significant decreasing trends of incidence and mortality from cervical cancer has been registered in the last decades, thanks to the introduction of organized screening.^1^ Based on GLOBOCAN 2020 estimates, the highest rates of cervical cancer incidence and mortality are registered in developing countries, where a screening program is just being implemented or has not yet been introduced.^2^, ^3^, ^4^ From 2013, Serbia has implemented Papanicolaou smear testing for population screening for cervical cancer.^5^ In 2020, rates of incidence and mortality of cervical cancer in Serbia were among the highest rates in Europe.^2^ Every year in Serbia, around 1300 women get cervical cancer and about 500 women die from this disease.^5^

It is known that women may experience increased depression at the time of any cervical examination, particularly after receiving an abnormal Papanicolaou smear result when undergoing cervical cancer screening,^6^, ^7^, ^8^ but it is less known how additional diagnostic procedures affect depression levels.^9^, ^10^, ^11^ In the TOMBOLA study, Sharp and coauthors observed a cumulative prevalence of depression of 10.0% during the 30‐month follow‐up period after colposcopy and biopsy (7.9% before colposcopy and biopsy, and 6.6% after 6 weeks of these procedures); during the follow‐up period, a statistically significant increase in depression levels was observed after diagnostic procedures.^10^, ^12^ In contrast, the results of most of the other studies showed that depression was lower after colposcopy.^13^, ^14^, ^15^, ^16^ In a study that assessed depression in women with a mildly abnormal Pap smear just before undergoing diagnostic procedures, 28% of women were marked as significantly depressed.^17^ In contrast to these results, in women with an abnormal Papanicolaou screening test immediately before colposcopy and biopsy in one study in the United States, significant depressive symptomatology was observed in 64% of subjects.^18^

Some studies did not find significant differences in the level of depression of women referred for colposcopy and biopsy in relation to their age, level of education, and smoking, while some studies suggested an association with high state anxiety, worry about cancer, worry about having sex, and satisfaction with support.^19^, ^20^ But, most studies have been conducted in developed countries, while experiences from countries with limited resources are lacking or insufficient in the context of the burden that cervical cancer has in those populations. In order to improve the course of the disease, further research into predictors of depression before and after diagnostic procedures is of great importance for women with a positive Papanicolaou test, especially in countries where organized cervical cancer screening program is only just being introduced.

The aim of this research was to investigate the frequency and correlates of depression in women with positive Papanicolaou screening test before and after diagnostic procedures in Serbia.

## 2 METHODS

### 2.1 Setting

The study was conducted at the University Clinical Center Kragujevac, one of the four health facilities in Serbia that provide tertiary health care. The Clinic of Gynecology and Obstetrics at the University Clinical Center Kragujevac is one of the main health facilities responsible for the implementation of the national cervical cancer screening program in Kragujevac. At the Clinic of Gynecology and Obstetrics consultative colposcopy/biopsy/endocervical curettage are performed in women who were, within the screening program, found to have a positive cervical smear. The study was conducted in 2017.

### 2.2 Study design

A cross‐sectional study was carried out in a cohort of women with positive Papanicolaou screening test before and after diagnostic procedures (i.e., colposcopy/biopsy/endocervical curettage).

### 2.3 Study sample

The sample consisted of every consecutive woman attending cervical cancer screening, in whom, due to the positive result of the Papanicolaou screening test, additional diagnostic procedures were performed at the Gynecology and Obstetrics Clinic in Kragujevac.

**FIGURE 1:**
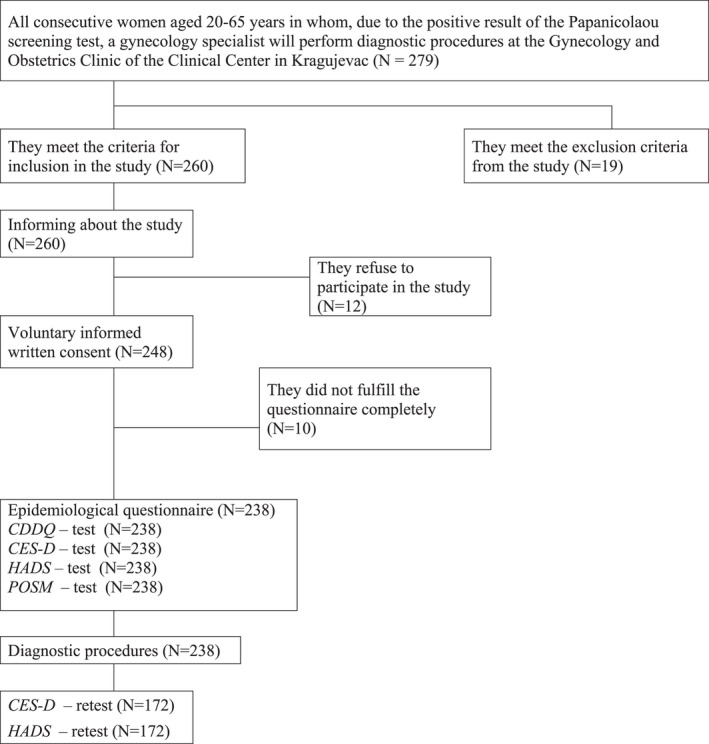
Research flow diagram

When women came for additional diagnostic procedures at the Clinic, 4–6 weeks after they had been informed about their last Papanicolaou screening test result, they received an invitation to participate in the study (Figure 1). Criteria for inclusion of participants in the study were: receiving a positive Papanicolaou test result and undergoing diagnostic procedures at the Gynecology and Obstetrics Clinic in Kragujevac, age 20 to 65, residency in the Kragujevac district area and fluency in spoken and written Serbian language, and voluntary informed written consent to participate in the study and absence of exclusion criteria. Criteria for exclusion of subjects from the study were: presence of previous cervical cancer or cervical intervention, age under 20 and over 65 years, pregnancy that occurred during the study, presence of psychiatric diseases, existence of diseases of reproductive organs treated during the study, refusal to participate in research or existence of any other objective reason that prevents or hinders participation in the study. Additional reason for ineligibility was refusal to participate in resurvey.

The initial response rate (before diagnostic procedures) was 96.0% (*n* = 238/248); 10 women returned an incompletely fulfilled questionnaire. Only completely fulfilled questionnaires are included in the analysis. The questionnaire was again given to all responders, 2–4 weeks after undergoing diagnostic procedures, that is before taking the results of the conducted examinations, resulting in a total response rate of 72.3% (*n* = 172/238). Despite the efforts to include all respondents in the repeated survey, 66 respondents were not surveyed, because they did not personally take the results of the pathohistological examination in the scheduled time. For this reason, these respondents were excluded from the analysis.

### 2.4 Sample size calculation

According to a study by Sharp and coauthors, prevalence of depression according to the Hospital Anxiety and Depression Scale (HADS), that is score ≥8 on the HADS depression subscale, in women who tested positive for cervical cancer was 7.9% before diagnostic procedures and 16.0% after diagnostic procedures.^10^ Using Fleiss's formula with continuity correction with two‐way testing and a first type error α of 0.05 and a desired study strength of 95% (second type error probability β = 0.05), it was determined that a minimum sample of 154 subjects was required. Sample size calculation was performed using Epi Info Version 7.2.0.1, Centers for Disease Control and Prevention, Atlanta, Georgia.

### 2.5 Data collection

With information on the objectives of the study and a form for voluntary consent to participate in the study, the questionnaire was enclosed. The invitation to participate in the study was always handed to the respondents by the same person (study staff / clinician). In addition to the invitation to participate in the study in which all the information about the study was written, the same person was available to provide any additional information to all respondents. Participants had approximately 20 (±5) min to complete the questionnaire using a self‐report pencil and paper survey.

### 2.6 Instruments

In addition to the epidemiological questionnaire (invalidated questionnaire on sociodemographic characteristics, habits, reproductive characteristics, personal and family health history, etc.), the following measurement instruments (validated) were used in the research:

- Questionnaire for the assessment of anxiety and depression: “Hospital Anxiety and Depression Scale” (HADS)^21^;
- Specific questionnaire for the assessment of psychological distress in subjects in cervical cancer screening: “Psychological distress in cervical dysplasia” (Cervical Dysplasia Distress Questionnaire, CDDQ)^22^;
- Specific questionnaire for the psychosocial status of respondents in cervical cancer screening applied in the study “Trial of Management of Borderline and Other Low‐grade Abnormal Smears, TOMBOLA”, that is Specific Process and Outcome Specific Measure (POSM) scale.^23^

A sociodemographic questionnaire (invalidated) was used to obtain data about participants' age (≤50 / >50), place of residence (Rural / Urban), education level (≤8 years / >8 years), occupation (Housewife / Laborer / Farmers / Clercs / Professional), and marital status (With partner / Without partner). Also, survey included data about religious affiliation (Orthodox / Other), knowledge of the meaning of the term “dysplasia” or “precancerous” (Yes / No), family history of cervical cancer (No / Yes), family history of other gynecological cancers (No / Yes), family history of other cancers (No / Yes). Family history for cervical cancer, other gynecological cancers, and other malignancies was asked for grade I kinship, which included parents, children, and siblings. Also, survey included data about age of menarche (<14 years / ≥14 years), regularity of menstruation (No / Yes), menopause (No / Yes), age of menopause (< 56 years / ≥ 56 years), pregnancy (Ever / Never), age of first pregnancy (<21 years / ≥21 years), number of pregnancies (<3 / ≥3), abortion history (No / Yes), number of abortions (<3 / ≥3), spontaneous abortion (No / Yes), induced abortion (No / Yes), children (No / Yes), number of children (<3 / ≥3), number of sexual partners (<3 / ≥3), oral contraceptive use (Ever / Never), tobacco use (Ever / Never), alcohol use (Ever / Never), Body Mass Index (< 25 kg/m^2^ / ≥ 25 kg/m^2^), venereal diseases in personal history (Yes / No), and history of chronic diseases (Yes / No).

HADS is a self‐assessment scale used as a screening method to detect symptoms and assess the degree of depression and anxiety during the last week.^21^ HADS is a scale of 14 questions: seven relate to anxiety (HADS‐A) and seven to depression (HADS‐D). For the purposes of this study, a dichotomy of classification of respondents was conducted: a score of eight or more in HADS subscales indicates elevated levels of anxiety and / or depression, and a score of less than eight indicates low levels of anxiety and / or depression. A systematic review of the literature by Bjelland and coauthors showed that in most studies an optimal balance between sensitivity and specificity was achieved when the case was defined by a score of eight or more on HADS‐A and HADS‐D.^24^

The CDDQ questionnaire is a specific questionnaire for self‐assessment of psychological distress in women with a positive Pap smear in the previous year.^22^ The questionnaire has 23 questions and four domains: domains “Tension and discomfort” and “Embarrassment” measure psychological distress associated with medical procedures (colposcopy), and domains that measure the distress associated with the consequences of receiving an abnormal Pap smear involve concerns about “Sexual and reproductive consequences” and “Health consequences”. According to the available literature, the first study of the validation of the CDDQ scale in Serbian language was conducted within this research.^25^

POSM is a specific questionnaire for assessing the psychosocial status of respondents in cervical cancer screening considering the receipt of a positive result of a cervical cancer screening test.^22^ The questions refer to the period between receiving the abnormal result and completing the questionnaire. Recent results of the validation of the POSM questionnaire, using exploratory factor analysis, report the extraction of two factors: Factor 1, which contains four particles associated with Worry; and Factor 2, which contains three particles related to Satisfaction with information / support. Factor 1 had good reliability (Cronbach's alpha = 0.769), but the reliability of factor 2 was weaker (0.482).^26^

Questionnaires were freely available or the license was obtained directly from the current owner. The linguistic adaptation and validation of all questionnaires, based on internationally accepted methodology, were performed before the beginning of this research. In our study, the Serbian versions of all used scales (HADS and CDDQ) were valid and reliable instruments for the assessment of psychological effects among women with abnormal Papanicolaou smear results.^25^, ^27^

### 2.7 Statistical analysis

Statistical analysis included Chi‐Square test for categorical variables, and Wilcoxon rank test. Univariate and multivariate logistic regression analysis were used in the evaluation of variables that could be predictors of depression. Univariate logistic regression was used to determine the Odds Ratio (OR) with a 95% confidence interval (Confidence Interval, 95% CI) to assess the association between depression and selected characteristics of study participants. Multivariate logistic regression analysis was used to identify independent predictors of depression associated with undergoing additional diagnostic procedures in women with history of receipt of abnormal Papanicolaou test. The multivariate logistic regression model included all variables that were associated with depression in univariate analysis models at *p* < 0.05 values. All statistical analyzes were conducted using the SPSS (version 20.0, Chicago, IL). A significance level of *p* < 0.05 was defined for all tests.

## 3 RESULTS

Average age of our participants was 47.8 ± 11.1 years (range 23–65) (Table 1). More than half of the women were not informed about the meaning of the term “dysplasia” or “precancerous lesions”. A positive family history of cervical cancer was found in almost 10% of participants: in most women, the closest relatives (first degree kinship: mother, sister, or daughter) suffered from cervical cancer. Also, about 10% of women reported a positive family history for other gynecological malignant tumors, with the majority of cases reported in first‐degree relatives. In almost a third of the participants, data on positive family history were obtained for other malignant tumors, most often in close family members (mother, father, sister, and brother).

**TABLE 1:** Baseline characteristics of study participants (*N* = 172)

Variable	Number (%)
Age (>50 years)	75 (43.6)
Average age (Mean ± SD; Range)	47.8 ± 11.1; 23–65
Place of residence (Urban)	127 (73.8)
Occupation (Laborer/Housewife)	71 (41.3)
Education level (>8 years)	135 (78.5)
Total number of years of schooling (xMean ± SD; Range)	11.3 ± 2.7; 4–20
Marital status (With partner)	140 (81.4)
Religious affiliation (Orthodox)	168 (97.7)
Knowledge of the meaning of the term “dysplasia” or “precancerous” (No)	113 (65.7)
Family history of cervical cancer (Yes)	17 (9.9)
First‐degree relatives (Yes)	13 (76.5)
Mother	7 (53.8)
Daughter	1 (7.7)
Sister	5 (38.5)
Family history of other gynecological cancers (Yes)	18 (10.5)
First‐degree relatives (Yes)	17 (94.4)
Mother	14 (82.4)
Daughter	0 (0.0)
Sister	3 (17.6)
Family history of other cancers (Yes)	48 (27.9)
First‐degree relatives (Yes)	29 (60.4)
Mother, father	25 (86.2)
Daughter, son	0 (0.0)
Sister, brother	4 (13.8)

In our participants, frequency of symptoms that would indicate existence of depression is higher after diagnostic procedures (48.3%) than before diagnostic procedures (37.2%); difference in the frequency of depression before and after diagnostic procedures was statistically significant (*p* = 0.038) (Table 2). The mean overall score for the HADS‐D subscale of depression before and after diagnostic procedures (5.58 ± 4.21 and 6.36 ± 4.15, respectively) showed a statistically significant difference (*p* = 0.020). The median score on the HADS‐D subscale increased from 5.00 before diagnostic procedures to 7.00 after diagnostic procedures. The Wilcoxon rank test found a statistically significant increase in depression after diagnostic procedures (Z = −2.318; *p* = 0.020), with a small difference (*r* = 0.012).

**TABLE 2:** Depressive symptoms (by HADS‐D subscale) in women with abnormal Papanicolaou smear results, before and after diagnostic procedures

Depression	Before diagnostic procedures (*N* = 172)	After diagnostic procedures (*N* = 172)	*p*
	Number (%)	Number (%)	
HADS‐D subscale			
Depression (score)			
No (0–7 points)	108 (62.8)	89 (51.7)	
Yes (8–21 points)	64 (37.2)	83 (48.3)	0.038*
Mean ± Standard Deviation; Range	5.58 ± 4.21; 0–19	6.36 ± 4.15; 0–20	0.020**
Median	7.50	9.00	0.020**

Note: *p* (value by: *χ^2^‐test, **Wilcoxon rank test).

Abbreviation: HADS‐D, Hospital Anxiety and Depression Scale, Depression subscale.

For depression before diagnostic procedures, univariate logistic regression indicated the potential prognostic significance of older age, occupation, psychosocial status according to the POSM scale (for the Worry domain) and level of anxiety, while multivariate logistic regression indicated that age (OR = 1.60; 95% CI = 1.09–2.34; *p* = 0.017) and level of anxiety (OR = 1.61; 95% CI = 1.38–1.88; *p* < 0.001) were significant independent predictors of depression (Table 3). After diagnostic procedures, univariate logistic regression indicated the following significant predictors of depression: place of residence, psychological distress according to the CDDQ scale (for Tension and discomfort domain), psychosocial status according to the POSM scale (for Worry and Satisfaction with information / support domains), and level of anxiety. According to the results of multivariate logistic regression, significant independent predictors of depression were urban place of residence (OR = 0.12; 95% CI = 0.03–0.47; *p* = 0.002) and level of anxiety (OR = 1.85; 95% CI = 1.54–2.21; *p* < 0.001) (Table 4).

**TABLE 3:** Predictors of depression (by HADS scale) among women with abnormal Papanicolaou smear results, before diagnostic procedures

Variables	Univariate logistic regression			Multivariate logistic regression		
	OR	95% CI	*p*	OR	95% CI	*p*
Age (>50 years)	1.48	1.19–1.97	0.006	1.60	1.09–2.34	0.017
Place of residence (Urban)	0.55	0.27–1.08	0.084			
Occupation (Laborer/Housewife)	0.79	0.62–1.00	0.050	0.92	0.66–1.28	0.612
Education level (>8 years)	0.63	0.30–1.31	0.217			
Marital status (With partner)	0.89	0.41–1.94	0.773			
Age of menarche (≥14 years)	1.16	0.96–1.40	0.124			
Regularity of menstruation (Yes)	1.21	0.61–2.40	0.593			
Menopause (Yes)	1.63	0.87–3.05	0.131			
Age of menopause (≥56 years)	1.80	0.37–8.72	0.468			
Pregnancy (Ever)	0.60	0.22–1.61	0.306			
Age of first pregnancy (≥21 years)	0.85	0.42–1.73	0.659			
Number of pregnancies (≥3)	1.10	0.66–1.86	0.709			
Abortion history (Yes)	0.60	0.29–1.23	0.162			
Number of abortions (≥3)	1.24	0.77–2.00	0.367			
Spontaneous abortion (Yes)	0.99	0.44–2.24	0.975			
Induced abortion (Yes)	0.97	0.30–3.15	0.965			
Children (Yes)	0.43	0.16–1.13	0.086			
Number of children (≥3)	1.03	0.59–1.82	0.908			
Number of sexual partners (≥3)	0.79	0.54–1.14	0.199			
Oral contraceptive use (Ever)	0.54	0.22–1.36	0.191			
Tobacco use (Ever)	0.78	0.41–1.45	0.433			
Alcohol use (Ever)	0.68	0.29–1.58	0.370			
Body Mass Index (≥25 kg/m^2^)	1.08	0.57–2.02	0.822			
Family history of cervical cancer (Yes)	1.57	0.57–4.30	0.379			
Family history of other gynecological cancers (Yes)	1.80	0.68–4.80	0.240			
Family history of other cancers (Yes)	0.64	0.32–1.29	0.212			
Venereal diseases in personal history (Yes)	1.28	0.28–5.91	0.753			
History of chronic diseases (Yes)	1.56	0.74–3.31	0.245			
Knowledge of the meaning of the term “dysplasia” or “precancerous” (No)	1.08	0.57‐2.02	0.822			
Psychological distress by CDDQ subscales						
Tension and discomfort	0.92	0.55–1.52	0.738			
Embarrassment	1.08	0.78–1.51	0.645			
Sexual and reproductive consequences	1.23	0.75–2.04	0.413			
Health consequences	1.25	0.88–1.79	0.214			
Psychosocial burden by POSM subscales						
Worry	1.06	1.03–1.09	<0.001	1.01	0.97–1.05	0.612
Satisfaction with information/support	0.99	0.98–1.02	0.755			
High level of anxiety by HADS (score ≥8)	1.63	1.41–1.88	<0.001	1.61	1.38–1.88	<0.001

Abbreviations: 95% CI, 95% confidence interval; CDDQ, the Cervical Dysplasia Distress Questionnaire; HADS, Hospital Anxiety and Depression Scale; OR, odds ratio; *p*, probability; POSM, process and outcome specific measure.

**TABLE 4:** Predictors of depression (by HADS scale) among women with abnormal Papanicolaou smear results, after diagnostic procedures

Variables	Univariate logistic regression			Multivariate logistic regression		
	OR	95% CI	*p*	OR	95% CI	*p*
Age (>50 years)	1.04	0.80–1.34	0.788			
Place of residence (Urban)	0.12	0.05–0.27	<0.001	0.12	0.03–0.47	0.002
Occupation (Laborer/Housewife)	0.88	0.70–1.10	0.257			
Education level (>8 years)	0.98	0.47–2.03	0.957			
Marital status (With partner)	1.34	0.62–2.88	0.457			
Age of menarche (≥14 years)	0.99	0.83–1.18	0.909			
Regularity of menstruation (Yes)	1.48	0.75–2.90	0.257			
Menopause (Yes)	1.51	0.81–2.78	0.192			
Age of menopause (≥56 years)	2.27	0.41–12.59	0.350			
Pregnancy (Ever)	0.57	0.23–1.44	0.236			
Age of first pregnancy (≥21 years)	1.29	0.64–2.58	0.479			
Number of pregnancies (≥3)	1.39	0.84–2.32	0.206			
Abortion history (Yes)	1.71	0.83–3.52	0.148			
Number of abortions (≥3)	0.75	0.48–1.18	0.214			
Spontaneous abortion (Yes)	1.28	0.58–2.80	0.544			
Induced abortion (Yes)	0.27	0.07–1.04	0.057			
Children (Yes)	0.58	0.25–1.35	0.207			
Number of children (≥3)	1.51	0.85–2.66	0.157			
Number of sexual partners (≥3)	1.13	0.78–1.64	0.521			
Oral contraceptive use (Ever)	0.48	0.20–1.14	0.096			
Tobacco use (Ever)	1.18	0.64–2.15	0.598			
Alcohol use (Ever)	0.67	0.30–1.49	0.321			
Body Mass Index (≥ 25 kg/m^2^)	1.12	0.61–2.07	0.711			
Family history of cervical cancer (Yes)	1.61	0.58–4.43	0.362			
Family history of other gynecological cancers (Yes)	1.39	0.52–3.70	0.514			
Family history of other cancers (Yes)	0.88	0.46–1.70	0.705			
Venereal diseases in personal history (Yes)	1.45	0.32–6.69	0.633			
History of chronic diseases (Yes)	1.35	0.64–2.85	0.425			
Knowledge of the meaning of the term “dysplasia” or “precancerous” (No)	1.24	0.67‐2.28	0.495			
Psychological distress by CDDQ subscales						
Tension and discomfort	1.74	1.05–2.91	0.033	1.03	0.45–2.37	0.942
Embarrassment	1.12	0.81–1.55	0.505			
Sexual and reproductive consequences	1.36	0.83–2.23	0.221			
Health consequences	1.35	0.95–1.91	0.092			
Psychosocial burden by POSM subscales						
Worry	1.03	1.01–1.06	0.016	1.02	0.98–1.07	0.275
Satisfaction with information/support	0.97	0.95–0.99	0.024	0.99	0.96–1.03	0.652
High level of anxiety by HADS (score ≥8)	1.89	1.58–2.27	<0.001	1.85	1.54–2.21	<0.001

Abbreviations: 95% CI, 95% confidence interval; CDDQ, the Cervical Dysplasia Distress Questionnaire; HADS, Hospital Anxiety and Depression Scale; OR, odds ratio; *p*, probability; POSM, process and outcome specific measure.

## 4 DISCUSSION

Our study showed a significantly elevated level of depression associated with undergoing additional diagnostic procedures in women with a history of receiving an abnormal Papanicolaou test. Findings of this study suggest the role of older age, rural residence, and anxiety in shaping the risk of depression among the participants.

Despite decades of practices of organized program of cervical cancer screening in most developed countries, not enough is still known about the frequency and predictors of depression before and after diagnostic procedures among women with abnormal Papanicolaou smear.^6^, ^8^ In a study in the United Kingdom, which included women with low‐grade cytological abnormalities referred for colposcopy, the frequency of depression was 7.9% (using a score on the HADS scale of ≥8) immediately before performing diagnostic procedures, while at the end of the 30‐month follow‐up period, the frequency of depression was 16.0%.^10^ Another study in the UK, which included only women with a high degree of dyskaryosis, reported a significantly higher prevalence of depression (using the same score on the HADS‐D subscale) a week before the colposcopic examination was 23%, while a week after the colposcopic examination prevalence of depression was 20%.^28^ Also, in a study in Sweden, almost 30% of women with an abnormal Papanicolaou test had high levels of depressive symptoms immediately before colposcopy and biopsy, as well as 6 months after diagnostic procedures.^13^ In women with an abnormal Papanicolaou test result in a study in Thailand, depression (defined as HADS‐D score ≥11) was found in 1% of participants before colposcopy.^29^ On the other hand, some studies did not show an elevated level of depression before or after colposcopy in women with abnormal Papanicolaou test results in the Netherlands,^30^, ^31^ the UK,^16^ and Australia.^32^ The frequency of depression (37.2% before and 48.3% after diagnostic procedures) in our study was slightly higher than in similar studies, which may be related to different cutoff values for the same applied scales (cutoff value on HADS‐D subscale in our study was ≥8), with different intervals for measuring depression before or after diagnostic procedures, but also with differences in the degree of abnormality of Papanicolaou smear of the participants included in the research. Possible explanations for different frequency of depression may be differences in the demographic, epidemiological, reproductive characteristics of the respondents. Also, explanations of the difference in the frequency of depression include different experiences in the implementation of screening programs, in the availability of screening for cervical cancer, the application of a very wide range of measurement scales for the evaluation of depression, etc.

Some authors examined the impact of providing additional information to women referred for diagnostic procedures due to an abnormal cervical cancer screening test, thus examining the assumption that a woman's level of knowledge and information actually affects the level of depression.^11^, ^30^, ^33^ However, research in the Netherlands did not show statistically significant differences in the psychological distress of women who received additional educational information compared to women who received a standard set of information.^30^, ^31^ This spectrum of negative feelings includes anxiety and fear in general, as well as worry about the possible occurrence of cancer, the impact on sexual and reproductive life, etc.

Our results show that depression before diagnostic procedures is associated with older age and anxiety levels. Depression after diagnostic procedures was related to rural residence and level of anxiety.

Older age, as a predictor of depression before diagnostic procedures in our respondents, may be related to the higher prevalence of depressive disorders in older age, as well as to the presence of unrecognized anxiety disorders, or less emotional control in older women who have already passed experience of numerous adverse life events.^34^

The influence of rural residence as a predictor of depression after diagnostic procedures could be explained by association with some other, potential predictors that were not confirmed as significant in our study (such as awareness of Papanicolaou test results, and occupation indicated by univariate logistics regression). On the other hand, it is known that urban residence is characterized by easier access to health services, easier transport, better availability of information, as well as higher level of education, which is a possible explanation for the negative connection with depression after diagnostic procedures.^35^

It is known that a higher level of anxiety is a significant predictor of depression in women with an abnormal Papanicolaou screening test result before and after diagnostic procedures,^36^ which was confirmed in our study. Increased distress is probably due to some other factors that we have not examined, such as concerns about HPV infection, status of sexual partner regarding sexually transmitted diseases, etc.^37^, ^38^

Existing studies have been conducted mainly in developed countries, with different incidence of cervical cancer and experience related to cervical cancer screening, which significantly complicates the comparison of results. Besides, since the present study and others cited seem to have included women who are attending clinic and receiving guideline‐concordant care, consequently the research only included women who did participate in follow‐up protocols that probably led to an underestimation of women's depression.^39^, ^40^

The existence of depressive symptoms before, and persistence after diagnostic procedures, makes it necessary to identify the group of women at risk in order to provide adequate information and counseling to reduce psychological distress in women with a positive Papanicolaou smear screening test.^41^, ^42^, ^43^ Adequate adherence to diagnostic procedures would help to reduce the percentage of invasive cervical cancer, when survival is significantly lower than in earlier stages of the disease, and reduce treatment costs. This can be achieved by detecting and adequately controlling the predictive factors of depression in women with a positive Pap smear before and after diagnostic procedures, and through targeted and specifically targeted information that will be actively provided to these women.

To the best of our knowledge, our study is one of the few studies to examine depression in women with a positive Papanicolaou screening test before and after diagnostic procedures. For the assessment of outcomes, only validated questionnaires were used in our study. However, this study has several limitations, including the known shortcomings of the cross‐sectional study design, the use of self‐report questionnaires, the absence of clinical confirmation of depression, the inability to rule out information bias, the inability to rule out the influence of other factors (e.g., recent adverse life events or change in stressors) to which women may have been exposed in the meanwhile and which could affect the depression after diagnostic procedures. Also, this study did not provide data on other potential predictors of depression (such as socioeconomic status, concerns about HPV infection, sexual partner status regarding sexually transmitted diseases, etc.). Finally, the study did not determine the level of depression before the Papanicolaou screening test, which could further help clarify the impact of diagnostic procedures on depression.

## 5 CONCLUSIONS

Almost half of the women in our population experienced depressive symptoms while undergoing the cervical cancer screening, particularly those of older age, with rural place of residence and higher level of anxiety.

## CONFLICT OF INTEREST

No potential conflict of interest.

## AUTHOR CONTRIBUTIONS

II and MI equally contributed to this paper with conception and design of the study, data collection, data analysis, interpretation of the results, manuscript preparation, critical revision, and editing. GB and AD contributed to data collection. II, GB, AD, SSG, and MI read, reviewed, and approved the final manuscript.

## ETHICS STATEMENT

This study is a part of a research approved by the Ethics Committee of the Faculty of Medical Sciences, University of Kragujevac (Ref. No.: 01–2176), and by the Ethics Committee of the Clinical Center Kragujevac (Ref. No.: 01–2869). Voluntary written informed consent was obtained from each participant and confidentiality was protected.

## Data Availability

The data that support the findings of this study are available from the corresponding author upon reasonable request.
